# GENETICS OF PSORIASIS AND PSORIATIC ARTHRITIS

**DOI:** 10.4103/0019-5154.62751

**Published:** 2010

**Authors:** Vinod Chandran

**Affiliations:** *From the Division of Rheumatology, University of Toronto, Centre for Prognosis Studies in the Rheumatic Diseases, Toronto Western Hospital, Toronto, Canada.*

**Keywords:** *Copy number variation*, *environment*, *gene*, *human leucocyte antigen*

## Abstract

It is well established that psoriasis and psoriatic arthritis (PsA) have a strong genetic component. Recent advances in genetics have confirmed previous associations and new loci have been discovered. However, these loci do not fully account for the high heritability of psoriasis and PsA and therefore many genetic as well as environmental factors remain to be identified. This paper reviews the current status of genetic studies in psoriasis and PsA.

## Introduction

Psoriasis is a chronic immune-mediated inflammatory skin disease affecting 1-3% of the population.[1] Although generally not life threatening, it may be associated with important morbidity and disability.[1] About 30% of patients with psoriasis have psoriatic arthritis (PsA), defined as an inflammatory arthritis associated with psoriasis, usually seronegative for rheumatoid factor.[2,3] Both psoriasis and PsA are complex genetic diseases with a significant genetic component. The genetic basis of psoriasis and PsA is supported from evidence from family and twin studies, linkage studies, as well as population-based association studies. In this article, I have reviewed the current knowledge of the genetics of psoriasis and PsA.

Identifying genes underlying disease susceptibility involves a series of investigations beginning with familial aggregation studies, followed by segregation analysis, linkage analysis, association analysis and functional studies to identify and characterize genes.[4] For complex diseases, however, segregation and linkage analyses have not proven to be generally useful.[5–7] Therefore, for such diseases, once familial aggregation is demonstrated, it may be appropriate to proceed with association studies to determine susceptibility loci/genes. Once a gene is identified, functional studies in biological systems are required to characterize the gene(s).

However, it is important to clearly define the phenotype before embarking on a genetic investigation. Although, “psoriasis” has a varied presentation, e.g. chronic plaque psoriasis, guttate psoriasis, and palmoplantar psoriasis, most genetic investigations have been on patients with chronic plaque psoriasis. In addition, to decrease heterogeneity and to enrich the genetic component, most studies have focused on Type I psoriasis, i.e. psoriasis of onset before 40 year age.[8] Most studies on PsA have been on patients satisfying diagnostic criteria proposed by Moll and Wright, although research groups are now using the recently developed classification criteria for psoriatic arthritis (CASPAR) criteria.[3,9]

## Heritability of Psoriasis and Psoriatic Arthritis

Family and twin studies have clearly demonstrated that psoriasis has a strong genetic basis. Two large-scale epidemiological studies revealed a substantially higher incidence of psoriasis in relatives of patients with psoriasis compared to the general population. The recurrence risk for affected siblings (λ_s_) was estimated to be between 4 and 10.[10] Twin studies reveal a concordance rate for monozygotic twins to be between 62 and 70% compared to 21-23% for dizygotic twins.[11] A polygenic or a multifactorial pattern is the most likely mode of inheritance. The recurrence risk ratio for PsA is substantially higher than that for psoriasis. In the first study published in 1973, the prevalence of PsA among first-degree relatives of probands with PsA was found to be 5.5% compared to the calculated prevalence in the UK population of 0.1%.[12] The recurrence risk ratio for first-degree relatives (λ_1_) was 55, according to Risch's method.[13] In more recent studies, the λ_1_ for PsA was 30.4 and that for psoriasis was 7.6.[14] Strong heritability was also demonstrated in a recent study from Iceland.[15] The only twin study in PsA to date confirmed that genes are important for psoriasis but did not have the power to detect a genetic effect in PsA.[16] Thus, both psoriasis and PsA have a strong genetic component, and studies to unravel the genes underlying susceptibility are worthwhile.

## Linkage Studies

Linkage is the tendency for genes located nearby on the same chromosome to co-segregate and exists over a broad region. Linkage analysis aims to determine approximate chromosomal location of genes by looking for evidence of co-segregation with other genes whose locations are already known. Linkage analysis uses relatively few markers (400-800) for whole genome analysis and linkage regions determined are large (order of 20-40 centiMorgans) and of low resolution. Moreover, for a linkage analysis, family data with multiple affected family members must be available. Linkage analyses have been successful in identifying genetic basis of many diseases such as Huntington's disease, Alzheimer's disease, some forms of breast cancer, but have been less successful in identifying genes for common complex diseases. A number of psoriasis susceptibility loci have been mapped using linkage methods. These include psoriasis susceptibility locus (PSORS)1 on 6p21.3 [gene candidates- Human leucocyte antigen (HLA)-Cw6, CDSN, HCR, HERV-K, HCG22, PSORS1C3, OTF3, TCF19, CCHCR1, LMP, SEEK1, SPR1], PSORS2 on 17q [RUNX1, RAPTOR, SLC9A3R1, NAT9, TBCD], PSORS3 on 4q [IRF-2], PSORS4 on 1q21.3 [Loricrin, Filaggrin, Pglyrp, S100 genes within epidermal differentiation complex], PSORS5 on 3q21 [SLC12A8, Cystatin A, Zn finger protein 148], PSORS6 on 19p [JunB], PSORS7 on 1p [PTPN22, IL-23R], PSORS8 on 16q [CX3CL1, CX3R1, NOD2/CARD15], PSORS9 on 4q28-32 [IL-15] and PSORS10 on 18p11.[17–26] The strongest association is with a locus within the major histocompatibility complex (MHC) on chromosome 6p21 (PSORS1). With regard to PsA, only one genome-wide linkage scan has been conducted.[27] This study identified a locus on 16q close to the PSORS8 locus identified for psoriasis, but only when conditioned on paternal inheritance. Thus, based on linkage studies, promising loci have been identified for psoriasis.

## Association Studies

Genetic association is a statistical relationship in a population between an individual's phenotype and their genotype at a genetic locus. Association exists over a narrow range. The markers used must be in proximity to a disease susceptibility locus. Typically, in a genome-wide association analysis hundreds of thousands of markers are used. This type of analysis can use isolated cases and unrelated individuals as controls, and is thus more feasible. Association studies have far greater power, even if one needs to test every gene in the genome compared to linkage studies that have limited power to detect genes of modest effect.[27] Thus, association studies are currently popular means of studying the genetics of complex diseases.

A large number of association studies based on a candidate-gene approach have been carried out to identify genes underlying susceptibility to psoriasis and PsA. Since the PSORS1 locus within the MHC region on 6p provides the strongest linkage with psoriasis in genome-wide linkage scans, candidate genes in this region have been investigated. This gene dense region codes for a number of genes important in the immune response, including HLA and non-HLA genes.

Psoriasis was found to be associated with HLA Class I antigens in the 1970s and subsequently confirmed by molecular methods. Initially, association with HLA-C, specifically HLA-Cw6, and several HLA-B alleles was described. The association with HLA-B was later determined due to linkage disequilibrium with HLA-C.[28] HLA alleles have also been associated with PsA. While HLA-B13, -B16, and its splits -B38 and -B39, B17 and Cw6 are associated with psoriasis, with or without arthritis, B27 and B7 are specifically associated with PsA.[29] Associations with Class I alleles are stronger with HLA-B than HLA-C alleles. The association of HLA-C with PsA was found to be due to association with early onset psoriasis, since no association was found in patients with PsA and late onset psoriasis.[30] Since almost all patients with PsA have psoriasis, it is unclear whether the HLA associations described above are with psoriasis or with PsA, or both. The HLA alleles that are specific to PsA are HLA-B27 and possibly B7, B38, and B39.

The MHC region on chromosome 6p has strong linkage disequilibrium spanning about 4 megabases encoding greater than 160 genes.[31] Other candidate genes in the proximal MHC Class I region have also been investigated since the precise identity of the PSORS1 determinant has remained elusive due to the existence of strong linkage disequilibrium within this region, where at least nine genes with possible biologic significance have been associated with psoriasis HLA-B, HLA-C, PSORS1C3, OTF3, HCR, SPR1, SEEK1, corneodesmosin (CDSN), and TNF-α.[32–38] Subsequently, a detailed analysis of genomic DNA sequences and recombinant haplotypes strongly suggested that HLA-Cw*0602 is the disease allele at PSORS1.[39] Genes in this region have also been investigated for association with PsA. MHC Class I-related (MIC) genes, 100 Kb centromeric to the HLA-B locus, have been associated with PsA.[40] A meta-analysis confirmed an association between TNF-α -238 polymorphism and PsA.[41] A recent fine mapping of the MHC identified an association of PsA with SNP rs1150735 that resides 1.5 kb upstream of the gene ring finger protein 39 that was previously shown to be associated with disease progression in acquired immunodeficiency syndrome.[42] Thus, the susceptibility locus for PsA may lie more centromeric to that of psoriasis, closer to HLA-B.

With advances in technology, the whole genome can now be interrogated using markers called single-nucleotide polymorphisms (SNPs). Association studies using this technology [Genome Wide Association Studies (GWAS)] are currently the most popular in identifying susceptibility genes for complex diseases. Interleukin (IL)-12B on chromosome 5q and IL-23R genes on chromosome 1p were identified using GWAS.[43] Using a 25,215 gene-centric SNP platform for discovery and follow-up tag SNP and sequencing, this study confirmed a reported psoriasis-associated SNP in the IL-12B 3' untranslated region (rs3212227) and found a second SNP (rs6887695) located 60 kb upstream. This study also identified two missense SNP in IL-23R associated with psoriasis, one of which (rs11209026, Arg381Gln) is also associated with Crohn's disease. The largest psoriasis GWAS on subjects of European ethnicity was recently published.[44] The study had a discovery cohort of 1409 cases and 1436 controls. A GWAS analysis using 438,670 autosomal SNPs and 2.5 million imputed SNPs was conducted and 21 SNPs in 18 loci were selected for replication. These 21 SNPs were tested for replication in a cohort consisting of 5048 cases and 5051 healthy controls. Evidence of association was found at 10 of these 18 loci and was particularly compelling at 7 loci (combined *P*-value <5 × 10^−8^). Initial comparison of case-control allele frequencies confirmed association at following established susceptibility loci: HLA-C (rs12191877, *P* = 4 × 10^−53^), IL-12B (rs2082412, *P* = 5 × 10^−10^) and IL-23R (rs2201841, *P* = 3 × 10^−7^). Other loci with confirmed association included IL-23A, TNF-α-induced protein 3 (TNAIP3), TNFAIP3-interacting protein 1 (TNIP1), IL-4, and IL-13. Thus, in addition to HLA-C, three pathways of psoriasis susceptibility were identified: TH17 pathway [IL-12B (encoding the p40 subunit of IL-23 and IL-12), IL-23A (encoding the p19 subunit of IL-23), IL-23R (encoding a subunit of the IL-23 receptor)], NFκB pathway (TNFAIP3, TNIP1), and TH2 pathway (IL-4, IL-13). However, association signals identified in this study accounted for λ_s_<1.35 (including 1.25 due to HLA). Thus, much of the familial component of psoriasis remains to be determined.

A large GWAS in a Chinese population to identify susceptibility variants for psoriasis using a two-stage case-control design was recently published.[45] In the first stage, a genome-wide association analysis in 1,139 cases and 1,132 controls of Chinese Han ancestry using Illumina Human 610-Quad BeadChips was conducted. In the second stage, top SNPs identified in the first stage was investigated: two independent samples of 5,182 cases and 6,516 controls of Chinese Han ancestry, and 539 cases and 824 controls of Chinese Uygur ancestry. In addition to the strong replication for two known susceptibility loci MHC (rs1265181, *P* = 1.93 × 10^−208^, OR = 22.62) and IL-12B (rs3213094, *P* = 2.58 × 10^−26^, OR = 0.78), a new susceptibility locus within the LCE gene cluster on 1q21(rs4085613, *P* = 6.69 × 10^−30^, OR = 0.76) was identified.

Association studies in PsA have identified a number of genes outside chromosome 6p including IL-23R, IL-1, and killer-cell immunoglobulin like receptor genes.[46–48] A formal GWAS on PsA has not yet been done. However, in the GWAS on subjects of European ethnicity described above, 1755 cases were known to have psoriasis with PsA and 3523 had psoriasis alone.[44] Thus, the authors were able to investigate association with PsA and differences between PsA and psoriasis alone. In this secondary analysis, three loci were associated with PsA when compared to normal controls (HLA-C, IL-12B, and TNIP1). There was a statistically significant difference between PsA and psoriasis alone at three loci (HLA-C, IL-12B and IL-23R). HLA-C and IL-23R were more strongly associated with psoriasis alone, and IL-12B with PsA.[44] A smaller GWAS identified a novel PsA (and potentially psoriasis) locus on chromosome 4q27 that harbors the interleukin 2 (IL-2) and interleukin 21 (IL-21) genes.[49]

Deletions, insertions, duplications, and complex multi-site variants collectively termed copy number variations (CNVs) are found in all humans and are functionally important.[50] Associations between higher genomic copy number for beta-defensin genes on chromosome 8, and LCE3B and LCE3C members of the late cornified envelope (LCE) gene cluster on 1q21 and psoriasis were recently demonstrated.[51,52] Interestingly, the deletion of LCE3C and LCE3B genes did not contribute to susceptibility to PsA.[53] Thus, LCE gene may be a “skin specific” genetic risk factor.

## Environmental Factors

A review of genetic susceptibility to a complex disease is incomplete without reviewing environmental risk factors. A number of environmental risk factors are associated with psoriasis. These risk factors include streptococcal pharyngitis (with guttate psoriasis), human immunodeficiency virus (HIV) infection, trauma, smoking, stressful life events, and obesity.[54–59] Patients with HLA-Cw*0602 have a higher incidence of streptococcal-induced flares and Koebner's phenomenon.[57] In PsA, associations with Rubella vaccination, injury sufficient to require a medical consultation, recurrent oral ulcers, moving house, fractured bone requiring hospital admission, and HIV infection have been demonstrated.[60,61] Smoking, psoriasis, and PsA have an interesting relationship. Smoking is a risk factor for the development of psoriasis and there is a dose-response relationship.[58] Interestingly, the time to development of PsA decreases with smoking prior to psoriasis onset, but the time to development of PsA increases with smoking after psoriasis onset.[62] IL-13 gene polymorphisms influence this relationship. In a recent study, it was shown that the minor alleles (rs1800925*T, rs20541*A, and rs848*A) are associated with protection from PsA versus controls.[63] No association with psoriasis was seen when PsA cases were excluded. However, smoking appeared to abrogate this protective effect.[63]

## Genetic Factors Affecting Disease Phenotype

Genetic variants affect disease phenotype in both psoriasis and PsA. HLA-Cw*0602 is associated with early onset psoriasis, higher incidence of guttate or streptococcal-induced flares, Koebner's phenomenon, more severe course, and is more likely to remit with pregnancy.[8,55,57,64,65] HLA-Cw*0602 is not associated with later onset of psoriasis, palmo-plantar psoriasis, nail or scalp involvement and is less frequent in patients with PsA.[8,55,65] In PsA, HLA-B27, Cw2, and DRw52 are associated with Axial PsA, and HLA-B38 and B39 with polyarthritis.[29] It has also been shown that HLA-B27 in the presence of HLA-DR7, HLA-B39 and HLA-DQw3 in the absence of HLA-DR7 are associated with progression of clinical damage, and that HLA-DR7, B22 alleles are “protective.”[66,67] HLA-DRB1 rheumatoid arthritis shared epitope as well as IL-4 I50V polymorphism have been shown to be associated with erosive PsA.[68,69] Patients with PsA carrying both HLA-Cw6 and HLA-DRB1*07 alleles have a less severe course of arthritis.[30]

## Conclusion

Thus, a number of susceptibility loci for psoriasis and PsA have been discovered, and the pace of discovery has accelerated. Loci influencing disease expression have also been described. However, these loci explain only a fraction of the heritability estimates and so many additional genetic loci as well as shared environmental risk factors remain to be identified. These anticipated discoveries will help us understand pathogenesis, identify drug targets, and help predict disease course and response to pharmacotherapy in psoriasis and PsA.
